# Veterinary Medical Society, University of Pennsylvania

**Published:** 1898-03

**Authors:** M. Jacob

**Affiliations:** Secretary


### VETERINARY MEDICAL SOCIETY, UNIVERSITY OF PENNSYLVANIA.

The meetings held during the month of February were very interesting and instructive. They differed from previous meetings in many respects.

Among the new members who joined the Society were: Dr. Robert

Formad, who was unanimously elected an honorary member, and Mr. Joseph Johnson, who was elected as a member of the Society.

Mr. A. Cunningham, the ex-Treasurer of the Society, made a report, and it was moved and seconded to accept the same and to extend a vote of thanks for the valuable service rendered the Society.

Mr. Hoopes was appointed as a committee to inquire into the matter of the Society certificates at Avil & Company's.

Mr. Newcomer was appointed to see to the binding of the magazines in the library which were presented to the Society by Prof. J. W. Adams.

It was moved and seconded not to send out any inquiry-sheets this year, but to let each member of the Senior Class have twelve of the inquiry-sheets.

Next in order was a mock trial. Mr. Horner was supposed to have sold a horse to Mr. A. Cunningham for $250, the purchaser claiming that the horse was not as represented, and brought suit against Mr. Horner for $500 damages. The participants consisted of the following: Judge of the Court, Prof. L. Pearson; attorney for plaintiff, Dr. C. J. Marshall; attorney for the defense, Dr. W. H. Hoskins; examining veterinarian, Dr. W. G. Shaw; expert examining veterinarian, Prof. S. J. J. Harger.

Witnesses for plaintiff: Mr. Kirby, Mr. Hoopes, Mr. Cornman. Witnesses for defence: Mr. Chesley, Mr. Chesney, Mr. Nolan. The jury consisted of: Mr. Miller, Mr. Jones, Mr. Newcomer, Mr. Taylor, Mr. Land, Mr. Repp.

The jury brought in a verdict of $300 damages in favor of the plaintiff against Mr. Horner.

After the trial the members adjourned to the assembly-room, where a light lunch and smoker awaited them.

At the last meeting of the Society Mr. Atwood B. Hoskins gave a very interesting talk on pigeons. He spoke of the characteristics of the different breeds. He exhibited several specimens to show to the Society the wonderful changes from the blue-rock dove, among which were seen the jacobin, tumbler, pouter, homing or Antwerp, carrier, and fantail pigeons. Mr. Hoskins is one of the best and most extensive breeders of pigeons in this country, and undoubtedly is well versed on the subject, as was shown by the way he answered all questions which were put to him after his lecture. The short time that Mr. Hoskins was with the Society was greatly appreciated, and it was suggested that he should give the Society a talk at least once every year.

M. Jacob,
Secretary.

